# Horseback riding injuries in Sweden: a nationwide register-based study of fracture incidence

**DOI:** 10.1007/s00590-025-04596-8

**Published:** 2025-12-10

**Authors:** Hannah K Eriksson, Hanna Wullimann - Ohlsson, Olof Wolf, Anders Brüggemann

**Affiliations:** Department of Surgical Sciences, Uppsala University, Uppsala, Sweden

**Keywords:** Horseback riding, fracture, Swedish Fracture Register

## Abstract

**Purpose:**

Horseback riding (HBR) presents a significant risk of injury, particularly fractures. While popular in Sweden, data on HBR-related fractures remains limited in its specificity regarding demographics and injury patterns. This study sought to delineate the distribution of HBR-related fractures in Sweden, considering sex, age, fracture site, injury mechanism, and seasonal trends.

**Methods:**

We conducted a retrospective observational study using Swedish Fracture Register (SFR) data from 2015 to 2022. All HBR-related fractures were included. Data on sex, age, fracture site, injury energy level, and injury date were analysed using descriptive statistics.

**Results:**

Some 5,453 fractures in 5,001 patients were included. Females accounted for 93% of cases. Upper extremity fractures were most prevalent, with wrist fractures representing the most common location (21%). Fractures of the forearm and humerus were most common among children, in contrast to adults, who presented with a wider array of fractures. High-energy mechanisms constituted 30% of fractures and were more often associated with injuries to the spine, pelvis, and femur. Fracture incidence peaked in May and September, coinciding with increased riding activity. Fracture incidence showed an age-dependent increase, exhibiting a bimodal distribution among females, with peaks near 10 and 55 years of age.

**Conclusion:**

In the Swedish context, HBR–related fractures predominantly affect the upper extremities and occur most frequently in female riders, with distinct seasonal and age-related patterns. Although females account for most cases, the absence of detailed demographic data on the active riding population limits the ability to calculate precise incidence rates.

## Introduction

For centuries, humans have domesticated horses for work and recreation. Recent decades have witnessed a pronounced shift in focus toward equestrian pursuits and recreational activities, especially within industrialized nations. In Sweden, approximately 20,000 persons of all ages engage in horse-related occupations annually [1]. The Swedish Equestrian Federation reports that horseback riding (HBR) is the second most popular youth sport in Sweden. In 2024, the organisation reported a membership of 151,000. Of which 91% were female; approximately 105,000 members actively participate in leisure riding or in riding schools. Approximately 15% were licensed to compete. The number of practitioners has remained constant over the past decade [1]. In comparison, data from the US Centers for Disease Control and Prevention reveal that over 30 million individuals in the USA engage in equestrian sports and leisure activities each year, highlighting the global appeal of HBR [2]. Females represent most individuals involved in HBR-related injuries, reflecting the overall demographics of riders, in which females comprise the majority [3–7]. Given the widespread participation, a clearer understanding of injury patterns in this group is essential. Despite the popularity and documented injury risks of HBR, comprehensive demographic data on HBR-related fractures in Sweden remains scarce. The Swedish Fracture Register (SFR), established and achieving nationwide coverage by 2021, provides a comprehensive dataset for fracture research, including detailed data on injury mechanisms and patient demographics. This study employed the SFR to clarify the demographic characteristics of fractures sustained during HBR in Sweden, focusing on patient sex and age, fracture distribution, and seasonal variation of HBR-related fractures.

## Patients and methods

This observational register study is based on data from the SFR, a web-based national quality register that holds information on surgically and non-surgically treated fractures since 2011 [8, 9]. Registration is done using a personal identification number unique to each Swedish citizen. Age and sex are thus directly imported into the register. The treating orthopaedic surgeon registers the date of the injury, injury mechanism, fracture(s) sustained by that injury, including classification of fracture according to the AO/OTA classification, and fracture treatment prospectively. Treatment is registered as non-operative or operative. Operative treatment is further divided into type, e.g., plate, screw or intramedullary nail fixation, arthroplasty, amongst others.

The national coverage in SFR has been 100% since 2021, but the SFR’s completeness varies for different fracture locations and active departments. The overall completeness for fractures sustained by adults was 60% in 2021 [10] compared to the National Patient Register.

### Patient selection

The study included all patients, irrespective of age, with HBR-related fractures recorded in the SFR, with injury dates between January 1, 2015, and December 31, 2022. Out of 5,489 fractures, 36 were excluded due to missing fracture classification. Ultimately, 5,453 fractures in 5,001 patients were analysed in the study.

### Outcome variables

Baseline variables were sex, age, date of injury, injury mechanism, fracture classification, and primary treatment as non-operative or operative. The injury mechanism was registered as low- or high-energy injury. Fracture classification in the SFR is mainly done using the 2007 AO/OTA classification [11].

This study categorised fractures by body part to describe their distribution across the following areas: hand, radius and ulna (forearm), humerus, scapula, clavicle, spine, pelvis, acetabulum, femur, tibia/fibula, and foot. Details regarding operative versus non-operative treatment were excluded, as they were considered irrelevant to the scope of the study and more related to fracture type than location. Children were classified as individuals < 18 years old, while adults were defined as those aged ≥ 18.

### Statistics

Descriptive statistics were used to analyse sex and age distribution, injury date, and fracture.

localisation, number of fractures per occasion, and high or low energy injury. Categorical variables were presented with frequency, and continuous variables with mean or median, depending on whether the data were normally distributed. Fracture distribution was analysed for the entire cohort based on sex and age (children and adults). Seasonal variation was assessed as injuries per month. No statistical tests for comparison were performed. R software was used for all analyses.

### Ethics

The study was approved by the Swedish Ethical Review Authority (Dnr 2022- 04355–01, date of.

issue 20 September 2022). We performed this investigation following the Declaration of Helsinki.

### Patient and public involvement

Patients and the public were not involved in the design, conduct, reporting, or dissemination plans of this research.

## Results

Some 5,453 fractures in 5,001 patients related to HBR were identified over the study period. Most fractures (93%) occurred in females. The median age at injury was 25 years for females and 44 years for males, with a combined mean age of 26. 64% of fractures (n = 3,488) occurred in adults, while 36% (n = 1,965) were found in children. Adult females accounted for 58% of all cases. Among children, the median age was 12 years, with girls being older than boys (12 years vs. 9 years). In adults, the median age was 41 years; 40 years in females and 50 years in males. 86% (n = 4,689) were single fractures sustained on a single occasion, while 12% (n = 670) involved multiple fractures during one event. An additional 2% (n = 94) represented fractures from separate incidents in the same individuals, indicating recurrent HBR injuries.

**Fig. 1:**
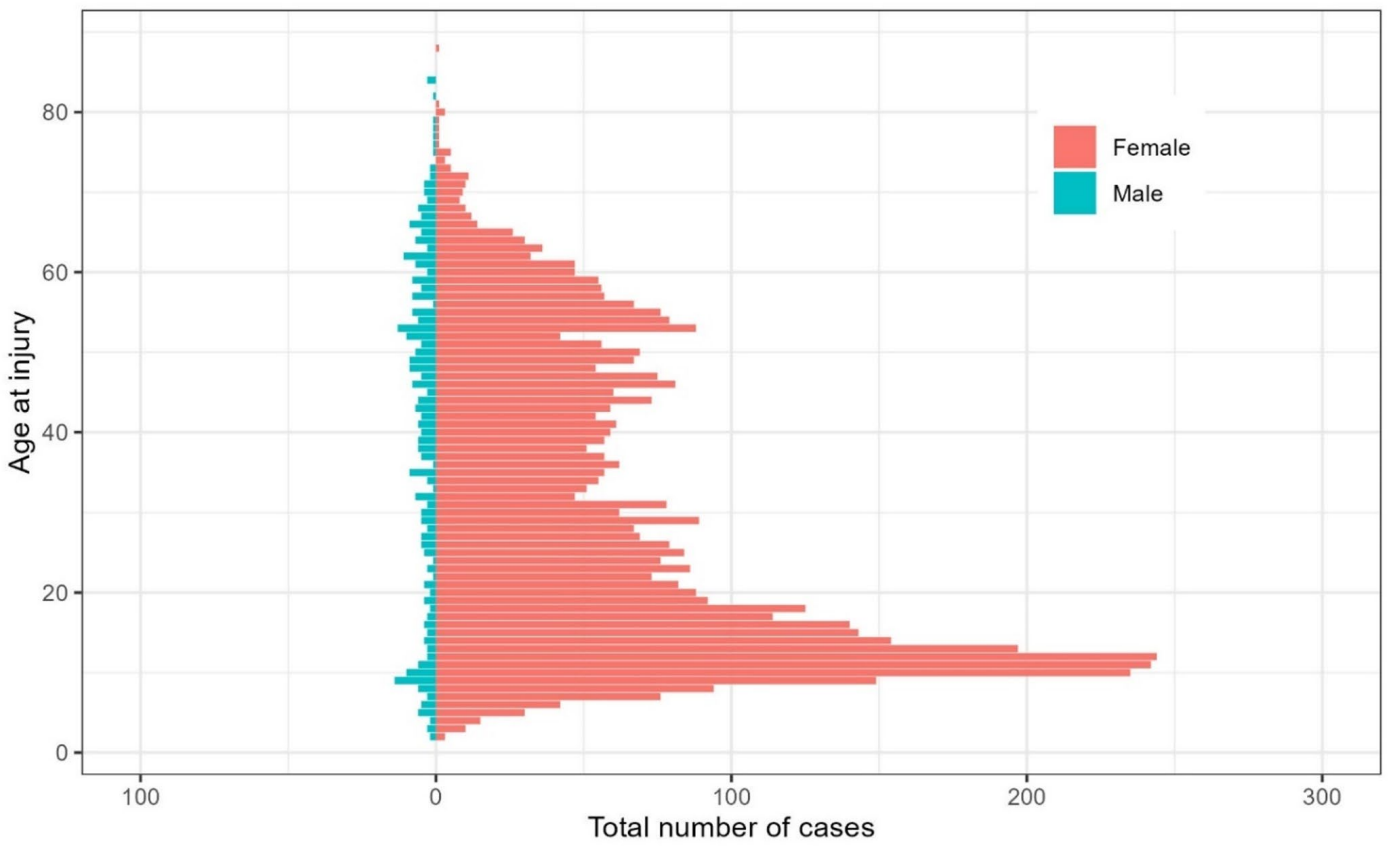
Sex and age distribution of 5,453 fractures sustained during horseback riding

In females, fracture occurrences displayed a bimodal age distribution, with peaks around ages 10 and 55, whereas in males, the distribution was more uniform throughout life (Fig. 1)

### Fracture distribution

Approximately 60% of fractures were in the upper extremity, 10% in the spine, 10% in the pelvis, acetabular or femur, and 20% in the tibia, ankle, or foot.

**Fig. 2:**
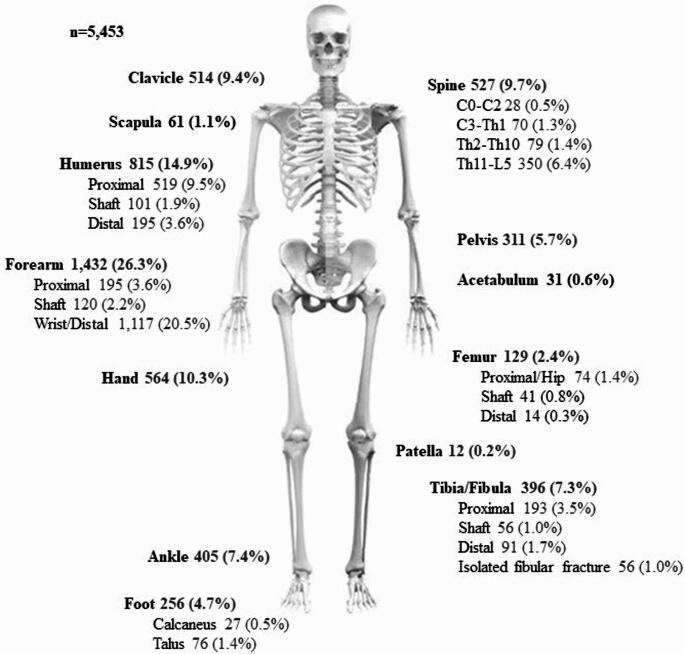
Fracture distribution of 5,453 fractures sustained during horseback riding

The distal radius was the most fractured site, accounting for 21% of all fractures (n = 1,117), followed by the humerus (15%), hand (10%), vertebrae (10%), and clavicle (10%) (Fig. 2).

The most frequently fractured bones in the hand were the fifth metacarpal, the proximal phalanx of the fifth finger, and the shaft of the ring finger. Among humeral fractures, the proximal segment was the most commonly affected, accounting for 10% of all fractures (n = 519). Similarly, the lumbar spine emerged as the most frequently fractured spinal segment, contributing 6% of all fractures (n = 350) and about two thirds of all spinal fractures.

**Fig. 3:**
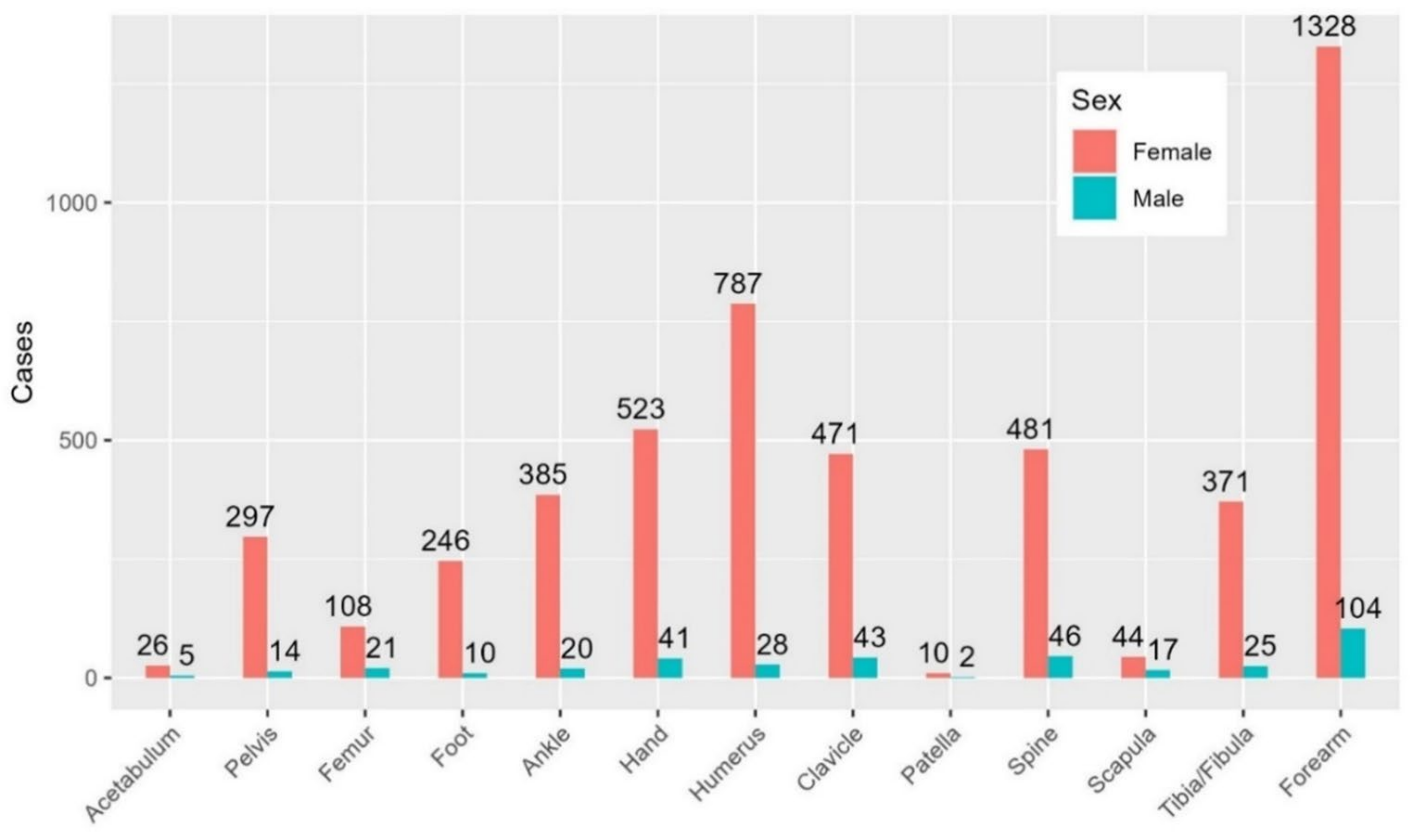
Fracture distribution by sex for all included fractures. Number of fractures on y-axis

The three most fractured areas in females were the radius and ulna, 26% (n = 1328), humerus, 16% (n = 787) and hand, 10% (n = 523) while in males, the radius and ulna, 28% (n = 104), vertebrae, 12% (n = 46), and clavicle, 11% (n = 43)) dominated (Fig. 3).

**Fig. 4:**
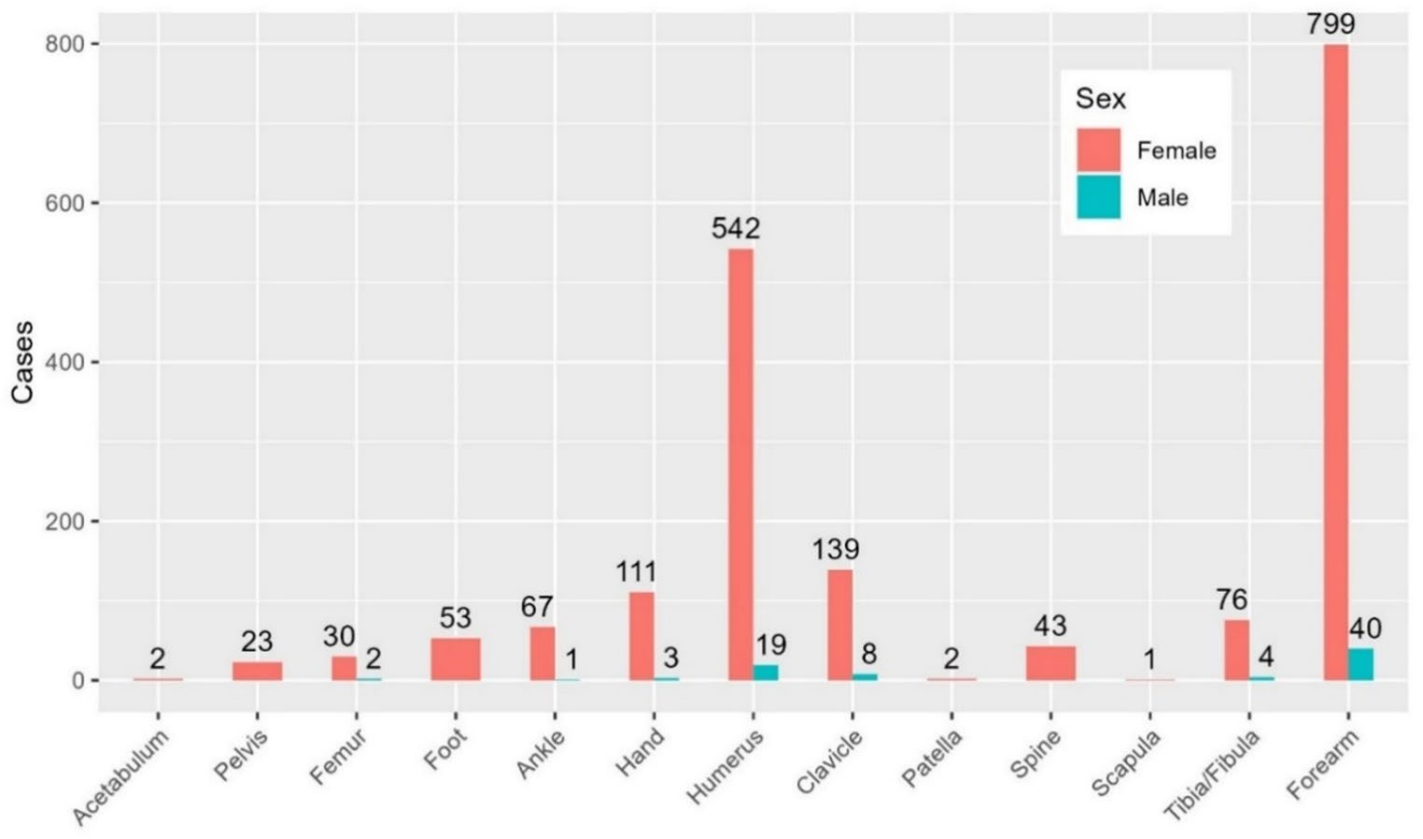
Fracture distribution in children, by sex. Number of fractures on y-axis

In children, the most common fracture sites for girls and boys were the radius and ulna, humerus, and clavicle. Fractures in radius and ulna were particularly predominant, making up 43% of fractures, followed by the humerus at 29% and the clavicle at 8% (Fig. 4).

**Fig. 5:**
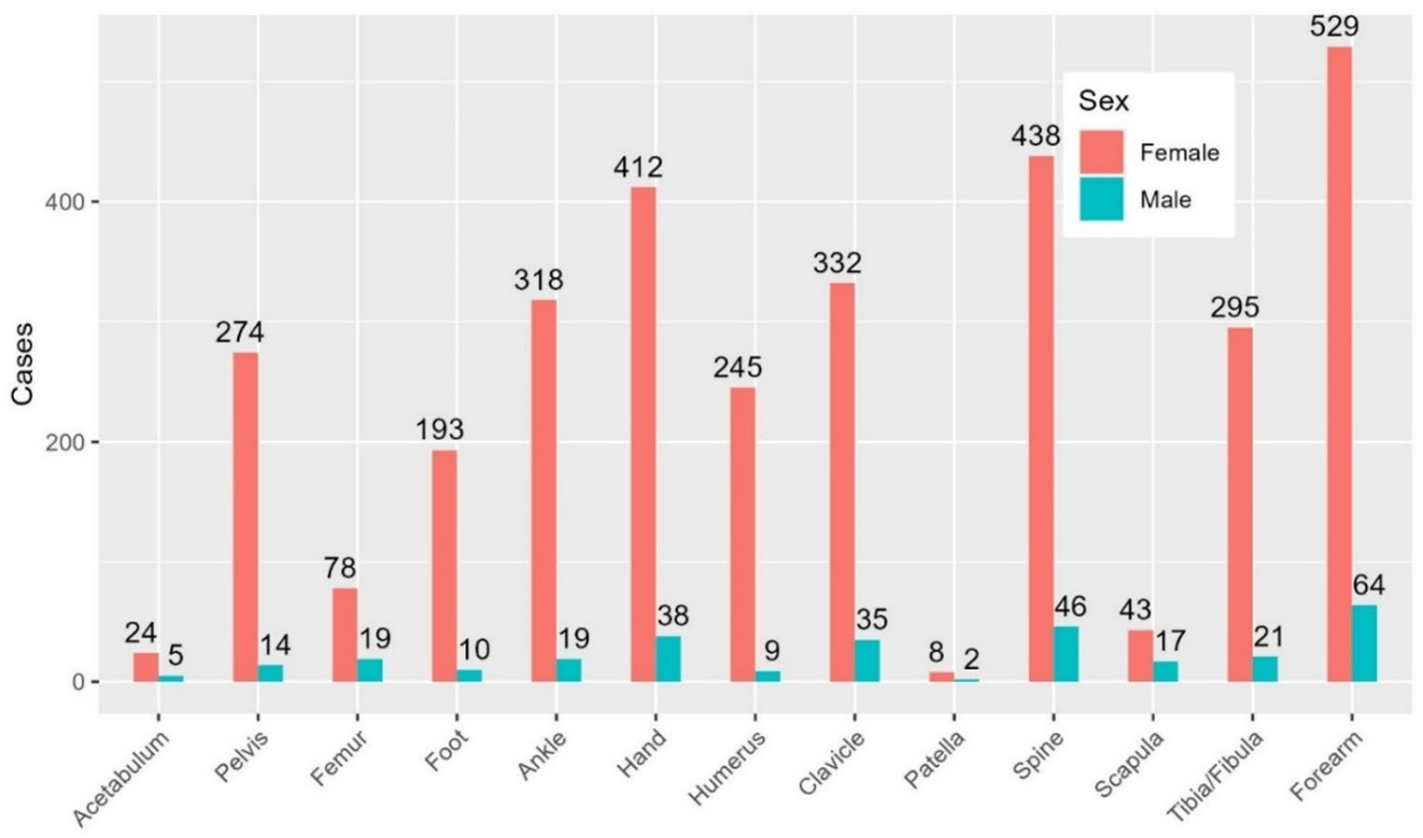
Fracture distribution in adults, by sex. Number of fractures on y-axis

Fractures in adults were distributed across a broader range of regions compared to those in children. In adults, fractures in radius and ulna comprised 17% of cases (n = 593), while vertebrae and hand fractures accounted for 14% (n = 484) and 13% (n = 450), respectively (Fig. 5). These locations were the top 3 for both females and males.

### Fracture characteristics by energy level

High-energy mechanisms were registered for 30% of fractures (n = 1,645). Nine per cent of the fractures had incomplete information regarding the energy level. The most frequent high-energy injuries occurred in the radius and ulna (20%), vertebrae (15%), and humerus (13%), whereas low-energy injuries primarily affected the radius and ulna (30%), humerus (17%), and hand (13%).

High-energy injuries were linked to a higher proportion of severe fractures in areas like the spine, pelvis, and femur. High-energy mechanisms contributed to 15% of vertebrae fractures, compared to 6% attributed to low-energy mechanisms. Only 2% (n = 109) were classified as open fractures. Among the open fractures, the tibia/fibula shaft was most commonly involved (32% of all open fractures), followed by the radius and ulna (24%) and ankle (21%).

### Seasonal variation

**Fig. 6:**
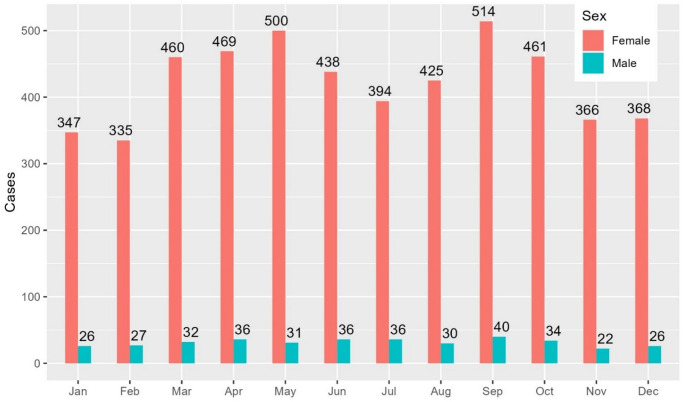
Seasonal variation for horseback-related fractures, by sex

Fractures demonstrated clear seasonal patterns, peaking in May and September. The highest number of fractures occurred in September, with 554 cases, while February saw the lowest, with 362 cases (Fig. 6).

## Discussion

Most fractures related to HBR occurred in the upper extremities, a pattern consistent with previous research highlighting the prevalence of upper limb injuries among equestrians [12]. In our study, fractures were more frequently observed among female riders, which likely reflects the greater proportion of females participating in HBR-related activities [3–6]. However, since detailed information on the gender distribution of participants is not available in this study, incidence rates could not be determined. A study by Stigson et al. reported an injury incidence of 10 per 1,000 female riders per year, compared to 4 per 1,000 male riders, indicating an almost threefold higher risk for females. In their cohort, 97% of the injured equestrians were women. Injury distribution also varied by age: 0.6% occurred in children aged 0–6 years, 13% in those 7–12, 26% in the 13–20 age group, 29% among individuals aged 21–40, and 31% in those over 40 [13]. Our findings are consistent with these results, including a similar age distribution and a mean rider age of 30.

In contrast, a U.S.-based study found that the most frequently injured equestrians were slightly younger, with females aged 15–19 representing the largest demographic presenting to emergency departments for HBR-related incidents [5].

Although fracture patterns were similar between sexes within each age group, distinct fracture sites were observed between children and adults. This suggests that age is more influential than sex in determining injury location.

In the overall cohort, the most commonly fractured regions were the radius and ulna, humerus, and hand, with the distal radius being the most frequently affected site. This is consistent with previous research emphasising the upper extremities’ susceptibility during falls, as individuals instinctively brace themselves [7, 9, 10]. The radius and ulna, and humerus were children’s most frequently fractured sites, whereas adults displayed various fracture locations. Children’s quicker reflexes may increase the likelihood of upper extremity injuries during falls [14].

Low-energy injuries were more common than high-energy injuries, with forearm fractures being the most frequent in both categories. High-energy injuries—often resulting from falls from significant heights or collisions—primarily affected the vertebrae and pelvis and were more prevalent in males, likely due to their higher participation in competitive or high-risk riding activities. Depending on height and impact conditions, falls from horses are generally considered high-energy events. Falls at full gallop can reach impact velocities of 30–50 km/h, with fall heights up to 3.5 m [15]. In contrast, low-energy injuries, typically associated with leisure riding, most commonly involve the radius and ulna, and humerus.

Seasonal peaks in fractures, occurring in May and September, likely reflect increased riding activity during favourable weather, while the lowest incidence in winter aligns with reduced outdoor riding. Exploring contextual factors such as riding location (indoors vs. outdoors) and weather conditions could provide insights into injury mechanisms. For example, outdoor riding may involve higher speeds, increasing the likelihood of high-energy incidents. Indoor riding may lessen injury severity due to both reduced speed and the presence of impact-absorbing footing materials, such as sand or fibre blends, which may reduce injury risk compared to outdoor terrain.

A study on HBR-related injuries identified three primary areas for prevention: rider safety, external factors, and rider-horse interactions [5]. Rider safety involves physical fitness, proper use of safety equipment, and avoiding riding alone. External factors include environmental conditions and the unpredictable nature of horses. To enhance rider safety, particular attention should be given to outdoor riding environments, such as trail riding and cross-country disciplines, which have been shown to carry a higher risk of severe skeletal injuries compared with indoor arenas due to environmental unpredictability and uneven terrain [16, 17]. Falls in these settings frequently result in serious trauma, including spinal injuries [16, 17]. Moreover, the British Horse Society’s national survey reported that 68% of riders experienced a near miss and 6% sustained an injury while riding on roads or off-trail routes, underscoring the dangers of uncontrolled environments [18]. Therefore, targeted safety measures—such as the consistent use of certified helmets and safety vests, pre-ride terrain assessments, weather-related precautions, and enhanced visibility requirements—should be prioritised to mitigate risk in outdoor equestrian activities. Addressing these areas through targeted interventions could significantly reduce the risk of injuries in horseback riding.

Future studies should encompass all HBR-related injuries, not just fractures, and include both mounted and unmounted incidents such as biting, kicking, trampling, and crushing. Additionally, qualitative research could explore riders’ perspectives on HBR-related injuries and practical and non-intrusive safety equipment. Carmicheal et al. highlighted that both mounted and unmounted riders are equally at risk of head injuries, with extremity fractures being common in both groups [19]. While the present study focuses on skeletal injury patterns, an important yet under-studied aspect is the psychological injury burden following equestrian trauma. Though the literature is scant regarding posttraumatic stress syndrome (PTSD) in recreational riders injured in horseback-riding accidents, evidence from equine-assisted therapy shows that horse-riding environments can ameliorate PTSD symptoms in veteran populations [20]. This suggests that the rider-horse-environment interaction has psychological import.

Future research should focus on exploring the long-term outcomes of HBR-related injuries and integrating SFR data with other national databases to provide a more comprehensive understanding of these injuries. While the SFR offers valuable insights into fractures, it does not capture head trauma or other common injuries. Bridging these data gaps could improve the understanding of HBR-related trauma and support the development of targeted prevention and rehabilitation strategies.

### Limitations

This study has several limitations related to data completeness and scope. Although the SFR achieved full national coverage of orthopaedic departments in 2021, this was not the case for the earlier years included in the study. Even with high coverage, not all fractures treated in these departments are registered, and completeness varies by fracture type and department. Non-operatively treated fractures, particularly common in paediatric cases, are less frequently registered, potentially biasing comparisons between children and adults. Certain HBR-related fractures, such as skull and rib fractures, were excluded from the analysis because they are not registered in the SFR. Injuries from unmounted incidents like biting, kicking or trampling were not analysed. Additionally, the absence of detailed data on Sweden’s active HBR population, including demographics, limits the ability to calculate incidence rates or compare the study population to the broader population. Further, the study does not capture long-term outcomes, such as recovery times, functional impairments, or quality of life following fractures.

Finally, as a retrospective analysis, this study relies on records that may be inconsistent or incomplete, particularly in capturing injury mechanisms and fracture severity. Improving the registration process for high- and low-energy injuries—potentially by including detailed options such as "still," "trot," or “gallop”—could provide greater accuracy and valuable detail.

Despite these limitations, the study’s use of SFR data offers a valuable and reliable foundation for tracking the prevalence and distribution of HBR-related fractures over time, particularly as preventive strategies evolve.

## Conclusion

Most HBR–related fractures occurred in the upper extremities and among females and younger individuals. Seasonal peaks suggest a link between riding activity and injury rates, underscoring the need for targeted prevention strategies.

## Data Availability

No datasets were generated or analysed during the current study.
